# Mapping the Site of Action of Human P2X7 Receptor Antagonists AZ11645373, Brilliant Blue G, KN-62, Calmidazolium, and ZINC58368839 to the Intersubunit Allosteric Pocket[Fn FN4]

**DOI:** 10.1124/mol.119.116715

**Published:** 2019-09

**Authors:** Anfal Bin Dayel, Richard J. Evans, Ralf Schmid

**Affiliations:** Department of Molecular and Cell Biology (A.B.D., R.J.E., R.S.) and Leicester Institute of Structural and Chemical Biology (R.S.), University of Leicester, Leicester, United Kingdom

## Abstract

The P2X7 receptor is a trimeric ligand-gated ion channel activated by ATP. It is implicated in the cellular response to trauma/disease and considered to have significant therapeutic potential. Using chimeras and point mutants we have mapped the binding site of the P2X7R-selective antagonist AZ11645373 to the known allosteric binding pocket at the interface between two subunits, in proximity to, but separated from the ATP binding site. Our structural model of AZ11645373 binding is consistent with effects of mutations on antagonist sensitivity, and the proposed binding mode explains variation in antagonist sensitivity between the human and rat P2X7 receptors. We have also determined the site of action for the P2X7R-selective antagonists ZINC58368839, brilliant blue G, KN-62, and calmidazolium. The effect of intersubunit allosteric pocket “signature mutants” F88A, T90V, D92A, F103A, and V312A on antagonist sensitivity suggests that ZINC58368839 comprises a binding mode similar to AZ11645373 and other previously characterized antagonists. For the larger antagonists, brilliant blue G, KN-62, and calmidazolium, our data imply an overlapping but distinct binding mode involving the central upper vestibule of the receptor in addition to the intersubunit allosteric pocket. Our work explains the site of action for a series of P2X7R antagonists and establishes “signature mutants” for P2X7R binding-mode characterization.

## Introduction

A receptor that is normally quiescent but becomes significantly activated in response to trauma/disease and exacerbates the condition would have considerable therapeutic potential. One such molecular target is the P2X7 receptor. The P2X receptor (P2XR) family comprises a number of cell-surface ATP-gated cation channels (Surprenant and North, 2009). The seven mammalian P2XR subunits (P2X1–7) assemble to form a range of homo- and heterotrimeric receptors with properties dependent on the subunit composition (Kaczmarek-Hájek et al., 2012). P2X7Rs can be distinguished from other P2XRs by their low ATP sensitivity, with an EC_50_ of approx. 0.3–1 mM at physiologic concentrations of divalent cations (Kaczmarek-Hájek et al., 2012). Normally, extracellular ATP levels are in the submicromolar range, and so P2X7Rs show limited basal activity (Di Virgilio et al., 2017). However, pathophysiological conditions (e.g., inflammation, cell damage, and necrosis) can result in high levels of extracellular ATP, leading to significant activation of P2X7Rs that are expressed on cells of hematopoietic origin as well as in glial, bone, epithelial, and endothelial cells (Kaczmarek-Hájek et al., 2012; Di Virgilio et al., 2017; Kaczmarek-Hajek et al., 2018). In addition, P2X7R expression levels can be upregulated in disease states, for example, epilepsy (Jimenez-Pacheco et al., 2013). In animal models, genetic knockdown and selective antagonists have demonstrated the contribution of P2X7Rs to a range of disease processes, including bone remodeling, cancer, inflammation, pain, transplant rejection, and several neurologic conditions (for reviews see Kaczmarek-Hájek et al., 2012; Burnstock and Di Virgilio, 2013; Bartlett et al., 2014; Bhattacharya, 2018; Cieślak and Wojtczak, 2018; Jimenez-Mateos et al., 2018). The human P2X7R exhibits a series of single-nucleotide polymorphisms that modulate receptor behavior. These P2X7R single-nucleotide polymorphisms are associated with several conditions, including pain sensitivity (Sorge et al., 2012) and bipolar disorder and depression (Skaper et al., 2010), supporting the therapeutic potential of P2X7R-selective antagonists in the treatment of human diseases.

A wide range of chemically distinct, highly selective P2X7R antagonists have been reported (Young and Gorecki, 2018). Crystallization and mutagenesis studies have identified an allosteric binding site for six of these P2X7R antagonists (Karasawa and Kawate, 2016; Allsopp et al., 2017, 2018). This allosteric site is at the subunit interface at the apex of the receptor (and referred to in this paper as the “intersubunit allosteric site”). Binding of these antagonists is thought to prevent narrowing of this pocket, thus also preventing the transition of the receptor into the open state (Karasawa and Kawate, 2016; Allsopp et al., 2017, 2018). In the P2X7R this pocket is larger than that seen in other P2XR-subtype structures, and the selectivity of the P2X7R antagonists is thought to arise from the size of the pocket and P2X7R-specific residue contacts with the compounds. In addition, residues in the pocket contribute to antagonist sensitivity but are conserved between P2XR subtypes. Analysis of the contribution of individual residues in the allosteric pocket has identified a series of residues associated with high P2X7R-antagonist potency (Allsopp et al., 2018).

There are several P2X7R antagonists whose sites of action remain to be established. AZ11645373 was first identified as a selective P2X7R antagonist by studies on recombinant human and native monocyte P2X7Rs and is effective in the nanomolar range (Stokes et al., 2006). AZ11645373 and the calcium-calmodulin–dependent protein kinase inhibitor KN-62 have been suggested to act at an allosteric site (Michel et al., 2007). These P2X7R-selective antagonists show species-dependent antagonist activity (more effective at the human P2X7R compared with rat) (Michel et al., 2008, 2009). Mutation to leucine of F95 at the human (h)P2X7R, the equivalent residue in rat (r)P2X7R, reduced antagonist sensitivity approx. 10-fold (Michel et al., 2008, 2009). On the basis of these observations, molecular docking proposed a binding site for AZ11645373 and KN-62 within the inner vestibule at the “top” of the extracellular portion of the receptor proximal to the orthosteric site (Caseley et al., 2015); this site is distinct from the allosteric pocket subsequently identified in the P2X7R crystallization and mutagenesis studies (Karasawa and Kawate, 2016; Allsopp et al., 2017). Like rP2X7R, the guinea-pig P2X7R has a leucine at position 95 but was more sensitive to KN62 than was the human receptor (Fonfria et al., 2008), which leads to questions about how far residue 95 accounts for differences in antagonist action among P2X7Rs in different species. In silico molecular docking has also suggested potential P2X7R orthosteric antagonist binding sites. The P2X7R antagonist ZINC 58368839 was identified by virtual ligand screening at the ATP binding pocket (Caseley et al., 2016), and AZ11645373 had a similar docking score in allosteric and orthosteric sites (Caseley et al., 2016). However, the modeled binding modes were not further validated by functional studies. For the P2X7R antagonists brilliant blue G (BBG) and calmidazolium a noncompetitive mode of action has been shown (Jiang et al., 2000; Stokes et al., 2006), but there is currently no specific information on the location of their binding sites. Thus the site of action of a range of P2X7R antagonists requires further investigation. In this study we have used chimeras and point mutants to map the binding site of AZ11645373. Further we have tested whether “signature” mutants in the P2X7R intersubunit allosteric pocket can be used to determine the site of action for the selective antagonists BBG, KN62, calmidazolium, and ZINC58368839 and have used ligand docking to explore in detail potential binding modes for these antagonists.

## Materials and Methods

### 

#### Pharmacological Characterization of P2X7Rs.

The P2X7-2N*β* chimeras and point mutants have been described previously (Allsopp et al., 2017). The rat P2X7R construct was kindly provided by Dr. Francois Rassendren, CNRS Montpellier, France. Point mutants were made using the QuikChange (Stratagene) mutagenesis kit. DNA sequencing (Automated ABI Sequencing Service; University of Leicester, UK) was used to confirm the mutation and absence of coding errors. The message mMachine (Ambion, Austin, TX) was used to make cRNA, and 50 nl (50 ng) was injected into manually defoliculated stage V *Xenopus laevis* oocytes with an Inject+Matic microinjector (J.A. Gabay, InjectMatic, Geneva, Switzerland). Oocytes were stored at 16°C in ND96 buffer (in millimolars, NaCl 96, KCl 2, CaCl_2_ 1.8, MgCl_2_ 1, sodium pyruvate 5, HEPES 5, pH 7.6) supplemented with 50 *μ*g/ml gentamycin and 50 *μ*g/ml tetracycline). Two electrode voltage-clamp recordings were made from oocytes bathed in divalent-free ND96 buffer (in millimolars, NaCl 96, KCl 2, sodium pyruvate 5, HEPES 5, and 0.1 flufenamic acid, pH 7.6) 3–7 days postinjection.

Oocytes were voltage-clamped at a holding potential of −60 mV with a GeneClamp500B amplifier. Currents were digitized with a Digidata 1322A and collected using pCLAMP 8.2 software (Molecular Devices, Menlo Park, CA). An EC_90_ concentration of ATP was used to test antagonist sensitivity for the P2X7-2N*β* and mutant receptors (ATP sensitivity of the chimeras and mutants are reported in Allsopp et al., 2017) to standardize for any changes in ATP sensitivity. ATP was applied via a U-tube perfusion system for 3 seconds. Antagonists AZ11645373 (Tocris), PPADS (Sigma), KN-62 (Adooq Bioscience), calmidazolium (Sigma), BBG (Alomone), and ZINC58368839 (Alomone) were bath-perfused as well as coapplied with ATP through the U-tube.

#### Molecular Modeling and Ligand Docking.

In preparation for ligand docking, five series of hP2X7-receptor–homology models were generated and ranked in MODELER using pdP2X7R-inhibitor–bound structures (Karasawa and Kawate, 2016) (PDB identifiers: 5U1U, 5U1V, 5U1W, 5U1X, 5U1Y) as templates. From each series the two best-scoring models were selected and prepared for RosettaLigand ensemble docking. The same approach was used to prepare rat P2X7R-homology models. RosettaLigand docking essentially followed the protocol outlined in Combs et al. (2013). The centers of allosteric and orthosteric docking sites were anchored at D92 and K64, respectively, with box-size parameters of 16 Å. The three-dimensional conformations for ligands were either downloaded from PubChem (Kim et al., 2019) or the ZINC library (Sterling and Irwin, 2015) or, when not available, generated in MarvinSketch 18.27, ChemAxon (https://www.chemaxon.com), on the basis of the SMILES tag for the respective ligand. For all compounds protonation states were analyzed in the Marvin pKA-plugin and hydrogens added accordingly. For each ligand multiple conformers were generated using the balloon software (Puranen et al., 2010) and merged into a multiconformer mol2 file. In total, 10,000 docking poses per docking site were calculated for each ligand, and the 10% best-scoring solutions were clustered as described previously (Allsopp et al., 2017, 2018). The clustering process resulted in representative solutions and distributions of Rosetta Interface scores, which were further analyzed for the main clusters. Models for representative docking poses (files: BBG_P2X7R.pdb, AZ11645373S_P2X7R.pdb, Calmidazolium_P2X7R.pdb, Zinc58368839_P2X7R.pdb and KN62_P2X7R.pdb) are provided as Supplemental Data (Data Supplements 1–5).

#### Data Analysis.

Inhibition by the antagonists was expressed as the percentage of the peak current amplitude to an EC_90_ concentration of ATP recorded before the application of antagonist (ATP gave reproducible responses in the absence of antagonist). Inhibition curves were fitted with the Hill equation (variable slope) using GraphPad Prism 6 (GraphPad Software, San Diego, CA). IC_50_ is the concentration of antagonist required to inhibit the response to an EC_90_ concentration of ATP by 50%. pIC_50_ is −log_10_ of the IC_50_ value. Individual concentration-response curves were generated for individual experiments, and statistical analysis was carried out on the data generated. When shown in figures the inhibition curves are fitted to the mean normalized data. Any significant differences from the P2X7-2N*β* control were calculated by one-way analysis of variance, followed by Dunnett’s test (using GraphPad Prism 6). Data are shown as mean ± S.D. In all cases *n* ≥ 3 for all data points.

## Results

### 

#### P2X7/1 Chimeras Suggest an Allosteric Binding Site for AZ11645373.

**Fig. 1.**
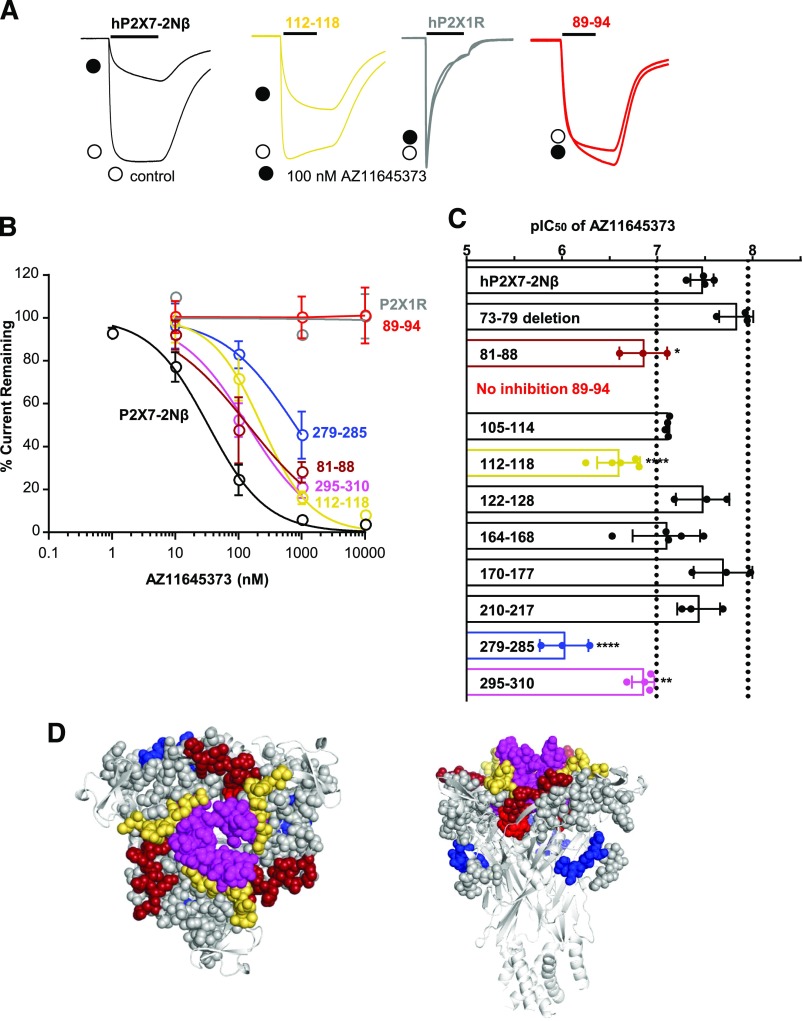
Chimeric hP2X7-1 receptors identify regions important for action of the P2X7R-selective antagonist AZ1165373. (A) Effect of the antagonist AZ11645373 (100 nM, traces indicated by black circle) on current evoked by an EC_90_ concentration of ATP (10-second application indicated by black bar) at the P2X7-2N*β*, 112–118, and 89–94 chimeras and P2X1R. Controls are indicated by open circles. (B) Concentration-dependent inhibition by AZ11645373 of responses to an EC_90_ concentration of ATP for P2X7-2N*β* (black), chimeras 81–88 (firebrick), 89–94 (red), 112–118 (yellow), 279–285 (blue), 295–310 (magenta), and P2X1 (gray). (C) Histogram showing the pIC_50_ of AZ11645373 at P2X7-2N*β* and chimeric receptors. Blacked dotted lines correspond to a 3-fold change in sensitivity. Exact values for each receptor tested are given in Supplemental Table 1. *P < 0.05; **P < 0.01; ****P < 0.0001, *n* ≥ 3. (D) Location of chimeras that reduced AZ11645373 action mapped on a pdP2X7R-based homology model; chimeras with no change are shown as gray spheres.

The hP2X7R shows current run-up/facilitation to repeated applications of ATP (Roger et al., 2010) that can complicate pharmacological analysis. We have previously shown that replacement of residues 16–26 of the intracellular amino terminus with those from the hP2X2R (the P2X7-2N*β* chimera) allows reproducible ATP-evoked responses to be readily recorded. The chimera has no effect on antagonist sensitivity and is therefore a useful background for studying the molecular basis of drug action (Allsopp et al., 2017, 2018). The P2X7R antagonist AZ11645373 inhibited ATP (100 *μ*M; EC_90_ concentration)-evoked currents at the hP2X7-2N*β* chimera in a concentration-dependent manner with an IC_50_ of approx. 30 nM (pIC_50_ of 7.5 ± 0.1) and was ineffective at the hP2X1R (Fig. 1), consistent with published work (Stokes et al., 2006).

To investigate the site of AZ11645373 action we tested the antagonist at a series of chimeras around the orthosteric and allosteric sites where the P2X7 residues were replaced by those from the P2X1Rs (using the P2X7-2N*β* background template). These chimeras (and point mutations around the allosteric site) have been described previously and, in combination with ligand docking, enabled validated molecular models of antagonist binding sites to be produced (Allsopp et al., 2017, 2018). To standardize the testing of antagonists at mutant receptors (chimeras and point mutants), we have used an EC_90_ concentration of ATP (for values see Allsopp et al., 2017, 2018). Chimeras that tested the contribution of a unique insertion (73–79) and deletion in the dorsal fin (chimera 210–217) of the P2X7R had no effect on AZ11645373 sensitivity (Fig. 1; Supplemental Table 1). There was also no change in AZ11645373 sensitivity for the chimeras 105–114, 122–128, 164–168, 170–177, and 210–217. Four chimeras that line the allosteric pocket showed decreased antagonist sensitivity (Fig. 1; Supplemental Table 1). There was a modest 4- to 8-fold reduction for the 81–88, 112–118, and 295–310 chimeras. At the 89–94 chimera (which corresponds to mutation of two unique threonine residues in the P2X7R T90V and T94V), AZ11645373 at 10 *μ*M had no effect on ATP-evoked currents. There was also an approx. 30-fold decrease in AZ11645373 sensitivity at the 279–285 chimera targeting the left flipper (forms part of the orthosteric pocket). However our previous work showed that this chimera also decreased sensitivity to the antagonist A740003, which binds at the intersubunit allosteric site (Allsopp et al., 2018). Replacement with the corresponding region from the P2X4 receptor had no effect on sensitivity (data not shown), suggesting this region is not involved in binding. The reduction therefore most probably results from an effect on the conformation of the intersubunit allosteric pocket through interaction of the 279–285 region with the *β*-strand Y291-K300, which separates the ATP binding site from the base of the allosteric pocket (Allsopp et al., 2018). Taken together these results for the chimeras suggest that AZ11645373 binds at the intersubunit allosteric site.

#### Combination of Mutagenesis and Ligand Docking Establishes the Site of AZ11645373 Action.

**Fig. 2.**
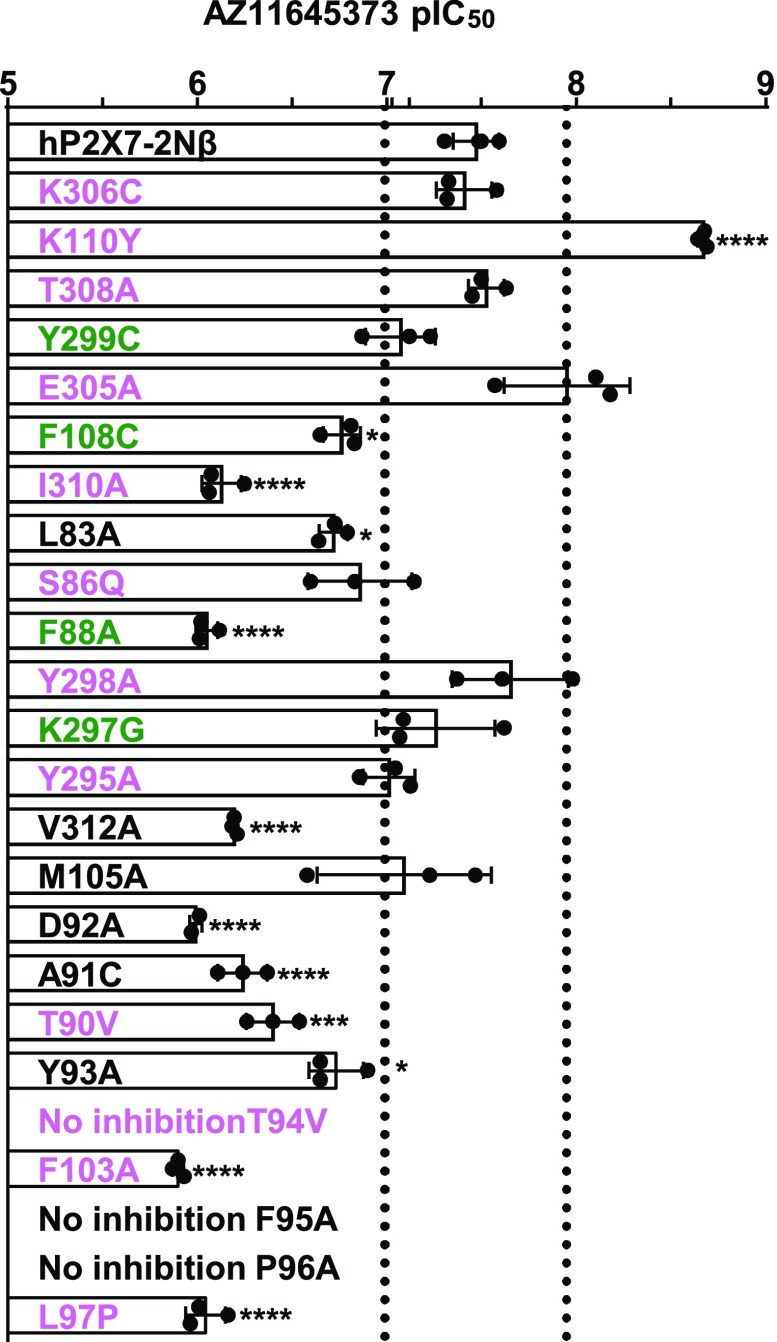
Effects of point mutants in the allosteric binding pocket on sensitivity to the antagonist AZ11645373. Effects of point mutations on AZ11645373 sensitivity are reported by their pIC_50_ value. Black dotted lines correspond to a 3-fold change in sensitivity. Pink residues are variant, green have similar properties, and those in black are conserved between P2X and P2X7Rs. Exact values for each receptor tested are given in Supplemental Table 32. *P < 0.05; ***P < 0.001; ****P < 0.0001, *n* ≥ 3.

The intersubunit allosteric pocket can be divided into the entrance, middle, and base. Point mutations of residues forming the pocket have been described previously and used in combination with molecular docking to provide models of antagonist binding sites (Allsopp et al., 2017, 2018). For amino acids different between the P2X7 and P2X1 receptors, the corresponding P2X1R residue was introduced. When the residue was similar/conserved between the two receptors, an alanine or cysteine mutant was tested (Allsopp et al., 2017, 2018). At the entrance to the allosteric pocket of the hP2X7R there are two positively charged lysine residues, K110 and K306, unique to mammalian P2X7Rs (human, rat, panda, rhesus monkey, and guinea-pig) (Fonfria et al., 2008; Bradley et al., 2011; Karasawa and Kawate, 2016). Removal of the positive charge had no effect at residue 306 (K306C), but at position 110 (K110Y) increased AZ11645373 sensitivity approx. 16-fold (*P* < 0.0001). Phe 108 (F108C) adjacent to Lys 110 on the beta strand (105–112) in contrast decreased AZ11645373 sensitivity approx. 5-fold. The L83A and S86Q mutants also reduced antagonist sensitivity by approx. 5-fold, consistent with effects of the 81–88 chimera. The mutations T308A, Y299C, and E305A had no effect on antagonist action. In the middle region of the pocket mutations, Y298A, K297G, Y295A, and M105A had no effect on AZ11645373 sensitivity. There was an approx. 20- to 25-fold decrease in antagonist sensitivity at I310A, and V312A (both facing the pocket from beta strand 306–312), F88A, and D92A (which removes the aspartic acid that is conserved in all seven mammalian P2XR subunits) reduced antagonist sensitivity approx. 25- to 30-fold. At the base region of the pocket all the point mutations showed decreased AZ11645373 sensitivity (Fig. 2; Supplemental Table 2). This was most pronounced for the T94V, F95A, and P96A mutants whose antagonist at 1 *μ*M had no effect on ATP-evoked currents, indicating a >1000-fold reduction in sensitivity. These mutagenesis results highlight residues in the intersubunit allosteric binding site that contribute to AZ11645373 action.

**Fig. 3.**
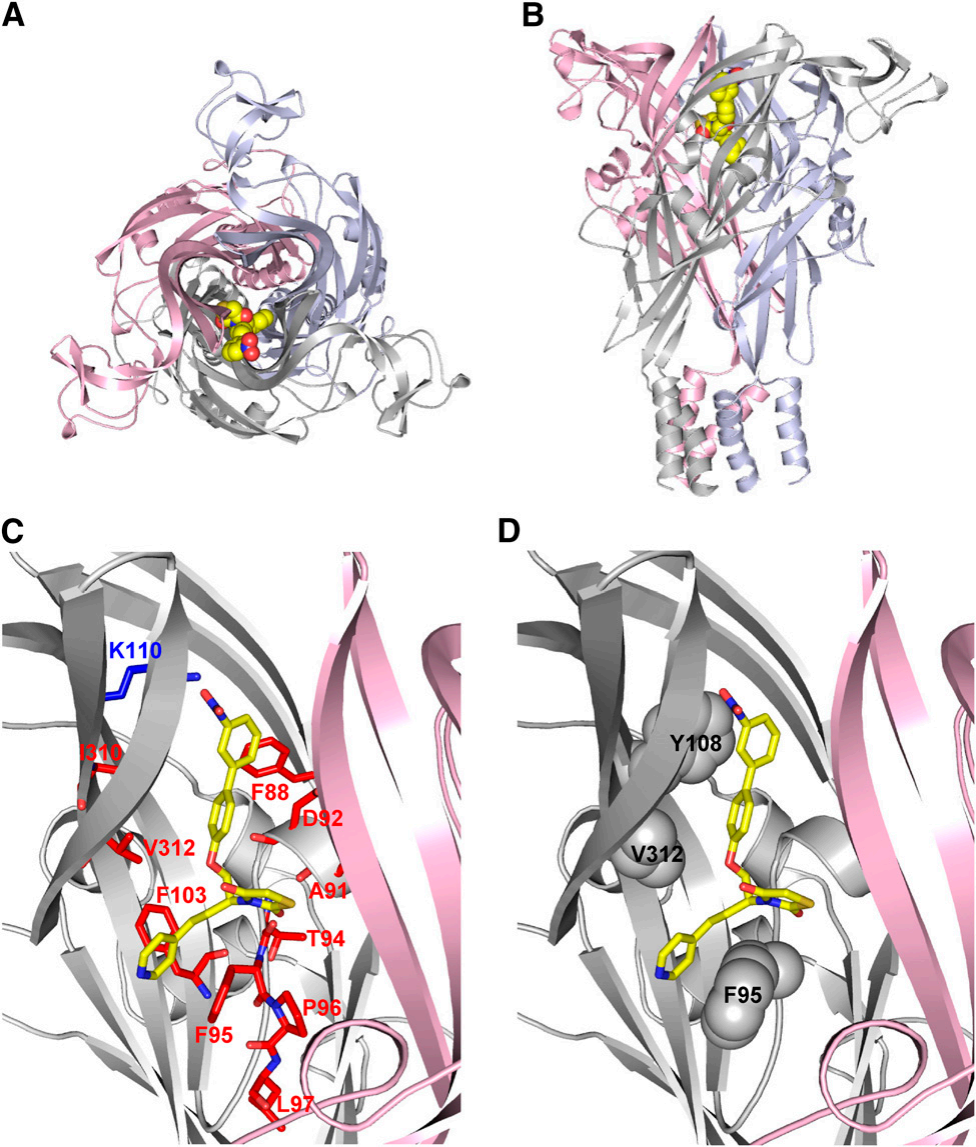
Representative binding pose for AZ11645373 in the hP2X7R. (A) View from the top of the extracellular domain along the central axis perpendicular to the membrane. The P2X7R model is shown as cartoon, with the three subunits highlighted in light blue, light pink, and gray; AZ11645373 is shown as spheres. (B) As in (A) but rotated 90°. (C) Zoom into the proposed AZ11645373 binding site, one subunit [light blue in (A) and (B)] is omitted for clarity. Residues K110 (blue, increase), I310, F88, V312, D92, A91, T94, F103, F95, P96, L97 (red, decrease or no inhibition), whose mutations showed the strongest effects on AZ11645373 sensitivity, are shown as sticks (data from Fig. 2). (D) As in (C), F95, V312, and Y108 residues, whose P2X7R rat-to-human and human-to-rat mutations (data shown in Fig. 4) affected AZ11645373 sensitivity, are shown as spheres.

Ligand docking was used to probe how AZ11645373 might bind to the hP2X7R. As it is not clear whether the *R* or *S* stereoisomer, or both, are the active entity in hP2X7R inhibition, both stereoisomers were used in docking. Although docking focused on the intersubunit allosteric binding site, both allosteric and orthosteric binding sites were sampled extensively in the docking process, with the orthosteric site serving as a decoy or null model. For both isomers, the means of Rosetta Interface docking scores for the biggest clusters (allosteric: −15.6 for *R*, −16.7 for *S*; orthosteric: −13.7 and −13.6) favor an allosteric binding mode over binding to the decoy, in agreement with the effects of selected mutations on antagonist potency (Supplemental Table 3). Also, on the basis of how the Rosetta Interface scores the *S*-isomer of AZ11645373, it is more probably the active compound. The representative pose for the major cluster of AZ11645373(*S*) places the nicotine and thiazolidinedione moieties deep in the allosteric pocket while the aromatic nitro-substituent is pointing toward the entrance of the pocket (Fig. 3) resembling the arrangements found in the pdP2X7R/antagonist X-ray structures (Karasawa and Kawate, 2016). Interestingly, this pose suggests that the side chain of K110 is involved in hydrogen bonding to the nitro group of AZ11645373. More favorable hydrogen bonding by the tyrosine hydroxyl group in the K110Y mutant might explain increased antagonist sensitivity. Residues in which a mutation has the biggest effects on AZ11645373 sensitivity line the proposed binding pose and are providing a hydrophobic environment for the core of AZ11645373. Notable are suggested aromatic interactions involving the nitrobenzene group of AZ11645373 and F88, and between the AZ11645373 nicotine group and F95 and F103 (Fig. 3). Almost all effects of point mutants on AZ11645373 antagonism can be rationalized in the context of the proposed binding pose. An exception is the L97P mutation: The L97 side chain is not in contact with the AZ11645373 docked model. This mutation might cause a more indirect effect on the shape of the allosteric pocket.

#### Introducing Sensitivity to the Rat P2X7R by Point Mutants of Species-Variant Residues in the Allosteric Pocket Validates the Model of AZ11645373 Binding.

**Fig. 4.**
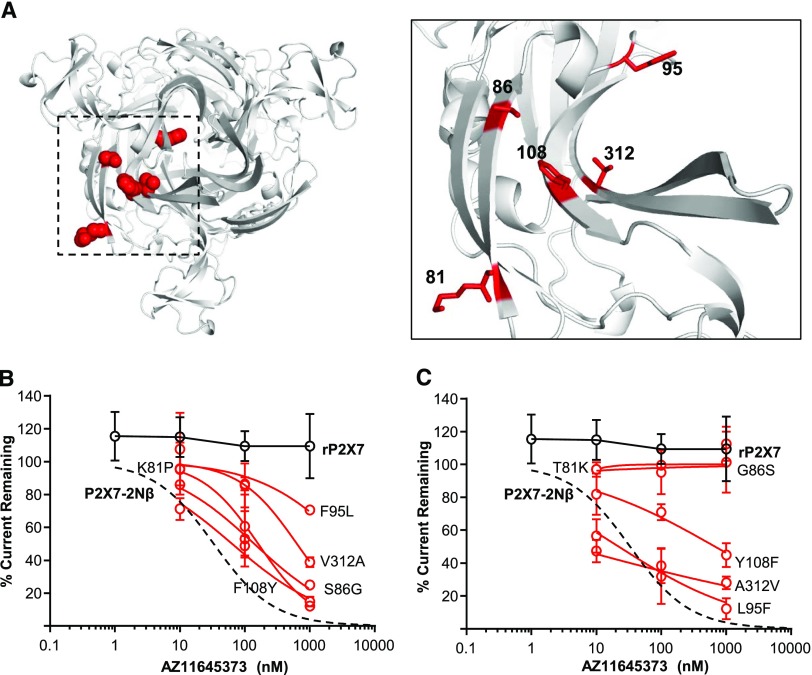
Introduction of AZ1165373 sensitivity by point mutation of species-variant residues in the allosteric pocket. (A) Homology model illustrating variant residues in the intersubunit allosteric pocket between hP2X7 and rP2X7R (red spheres) in the left panel, zoom and stick representation in the right panel. (B) AZ11645373 inhibition curves at human-to-rat P2X7R point mutations (red), P2X7-2N*β* (black dotted line), and rP2X7 (black). (C) AZ11645373 inhibition curves at rat to human P2X7R point mutations.

The combination of the chimeras, point mutations, and modeling provide a compelling model for AZ11645373 binding at the intersubunit allosteric site. One of the interesting features of AZ11645373 is that it is ineffective at the rat P2X7R (Stokes et al., 2006), and in the current study AZ11645373 (up to 1 *μ*M) had no effect on ATP-evoked currents (Fig. 4). If our intersubunit allosteric binding model is correct, then amino acid differences between the rat and human P2X7Rs should account for the variation in antagonist sensitivity. There are four variant residues between the human and rat P2X7Rs around the allosteric pocket: 86 at the entrance, 108/312 in the middle, and 95 at the base. We therefore made individual point mutations at the rat P2X7R, introducing the equivalent residue from the human receptor, and tested the effects of AZ11645373 (Fig. 4; Supplemental Table 4). At the L95F and A312V, rat P2X7R-mutant sensitivity to AZ11645373 was equivalent to that of the hP2X7R, the Y108F mutant had intermediate sensitivity (IC_50_ approx. 800 nM), and the T81K (in proximity to the entrance) and G86S mutants were not inhibited by the antagonist (up to 1 *μ*M). In reciprocal studies, the equivalent human-to-rat point mutations were tested. For the human-to-rat point mutations, there was an approx. 20- to 250-fold decrease in AZ11645373 sensitivity at the F95L and V312A mutants and little or no effect at the K81T and S86G mutants (Fig. 4; Supplemental Table 4). These results further validate the model of AZ11645373 binding to the intersubunit allosteric site. We also docked AZ11645373 into the intersubunit allosteric site of rP2X7R models. Rosetta Interface docking scores for the biggest clusters are consistently higher (indicating weaker binding) than found for docking into the intersubunit allosteric binding site of hP2X7R (Supplemental Table 3) in agreement with rat-to-human and human-to-rat point mutations.

#### Use of Signature Intersubunit Allosteric Point Mutants to Study Antagonist Action.

**Fig. 5.**
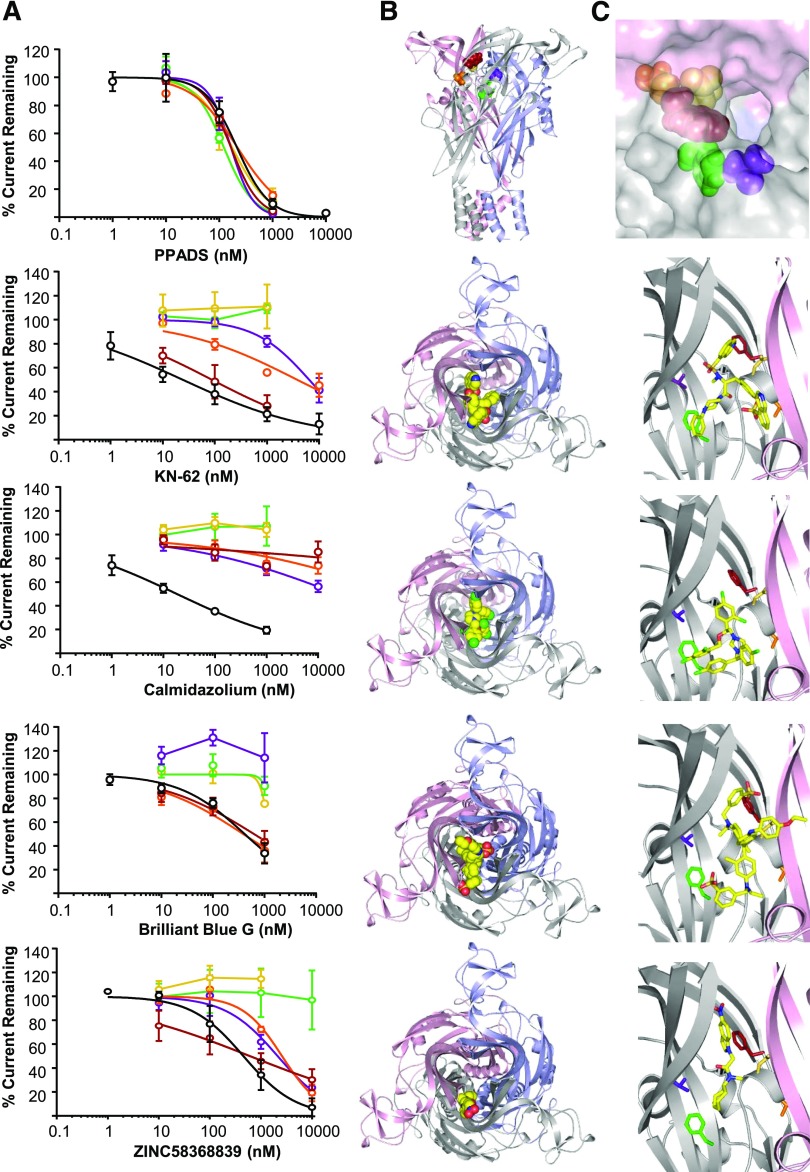
Use of intersubunit allosteric pocket “signature mutants” to investigate the site of action for brilliant blue G, KN-62, calmidazolium, ZINC58368839, and PPADS. (A) Concentration-dependent inhibition by P2X7 antagonists of response to an EC_90_ concentration of ATP for P2X7-2N*β* (black), F88A (firebrick), T90V (orange), D92A (yellow), F103A (green), and V312A (purple). (B) Top panel: P2X7R overview with “signature mutants” residues shown as spheres; colors as in (A). Other panels: View from the top of the extracellular domains along the central axis perpendicular to the membrane. The P2X7R model is shown as cartoon with the three subunits highlighted in light blue, light pink, and gray with the docked pose of the antagonist shown as spheres. (C) Top panel: Surface representation of entrance to allosteric pocket. Other panels: Zoom into the proposed binding site, one subunit [light blue in (B)] is omitted for clarity. Residues F88, T90, D92, F103, and V312 and the respective antagonist are shown as sticks [colors as in (A) and (B)].

The current study brings to seven the number of P2X7R antagonists that have been shown to bind at the intersubunit allosteric site. Comparisons of the effects of mutations of the residues lining the pocket for AZ11645373 (this study) and A740003, A438079, and AZ10606120 (Allsopp et al., 2018) show variations between the effects of point mutants for different antagonists. However, there is a consistent decrease in sensitivity at the mutations F88A, T90V, D92A, F103A, and V312A. These mutations may therefore provide a “signature” for an antagonist that binds at the intersubunit allosteric site. This was further supported by the finding that these mutations have no effect on the sensitivity to the nonselective P2X receptor antagonist PPADS (pIC_50_ = 6.7 ± 0.1) (Fig. 5) that binds at the orthosteric site (Huo et al., 2018). We therefore tested the effects of these five point mutations on the P2X7R-selective antagonists KN62, calmidazolium, brilliant blue G, and ZINC58368839 (Fig. 5; Supplemental Table 5). At the hP2X7-2N*β* chimera, KN62 and calmidazolium showed activity similar to the IC_50_s of approx. 30 and approx. 20 nM (pIC_50_ 7.7 ± 0.4 and 7.8 ± 0.2, respectively). We therefore tested the effects of these five point mutations on the P2X7R-selective antagonists KN62, calmidazolium, brilliant blue G, and ZINC58368839 (Fig. 5; Supplemental Table 5, Supplemental Table 6). Interestingly, for both these antagonists the Hill slope was shallow (0.4 ± 0.1 for KN-62 and 0.7 ± 0.1 for calmidazolium), indicating that they may have more than one binding mode (Supplemental Table 7). The P2X7R antagonists ZINC58368839 and brilliant blue G inhibited ATP-evoked currents at the P2X7-2N*β* receptor, with an IC_50_ of approx. 300 nM (pIC_50_s of 6.4 ± 0.2 and 6.4 ± 0.1, respectively). The most dramatic effect of mutations was seen for D92A and F103A, where antagonist activity of KN62, calmidazolium, brilliant blue G, and ZINC58368839 was essentially abolished. The V312A mutation followed a similar pattern, but its effect on antagonist activity was generally weaker compared with D92A and F103A; in particular, this is the case for ZINC58368839. However, the F88A and T90V mutations showed antagonist-specific effects. For instance, although F88A had no effect on sensitivity for KN62 and brilliant blue G, calmidazolium sensitivity was decreased by >1000-fold. The effect of the T90V mutation was similar to F88A, with the exception of KN62, where antagonist sensitivity was reduced by >100-fold. The finding that some mutations in and around the intersubunit allosteric binding site affect antagonist activity differentially implies they might be used to distinguish nuances of binding within the intersubunit allosteric pocket, and could be used to rationalize binding poses suggested from ligand docking.

#### Ligand Docking Provides Atomistic Models of KN-62, Calmidazolium, ZINC58368839, and Brilliant Blue G Action in Agreement with Mutagenesis Data.

To probe how far ligand docking on its own might provide evidence for binding to the intersubunit allosteric site and to identify plausible binding poses, KN-62, calmidazolium, ZINC58368839, brilliant blue G, and PPADS were docked at the allosteric binding pocket (using the orthosteric site as decoy), clustered, and analyzed (summarized in Supplemental Table 3). Indeed, docking scores for KN-62, calmidazolium, ZINC58368839, and brilliant blue G favor binding to an intersubunit allosteric site in agreement with the mutagenesis data described above. In full agreement with the non-effect of intersubunit allosteric pocket mutations on PPADS sensitivity, PPADS docking poses in both sites showed comparatively weak scores, rendering intersubunit allosteric docking poses improbable.

ZINC58368839, with a molecular weight of 329 Da, is roughly the same size as AZ11645373 and other previously characterized P2X7R antagonists (Karasawa and Kawate, 2016; Allsopp et al., 2017, 2018). In the representative docking pose of its main cluster, ZINC58368839 occupies the intersubunit allosteric binding site and roughly occupies the same space as AZ11645373 at the subunit interface at the apex of the receptor. The hydrophobic cycloheptane ring sits deeply in the intersubunit allosteric pocket and is aligned by F103 and V312, while the indole group is facing the entrance of the pocket and is in proximity of F88. One noticeable feature of the ZINC58368839 docking pose is that the K110 side chains are involved in hydrogen bonding of the nitro substituent on the indole group, a feature similar to the AZ11645373(*S*) docking poses. As for AZ11645373(*S*), such a binding pose would allow the binding of three ZINC58368839 molecules to the trimeric receptor in structurally equivalent binding sites.

Compared with AZ11645373 and ZINC58368839, the three antagonists KN-62, calmidazolium, and brilliant blue G are substantially bigger (almost twice the molecular weight). Considering this, it is difficult to envisage how they could occupy the intersubunit allosteric binding pocket in the same way. Owing to the bigger size and additional degrees of conformational freedom, ligand docking for these compounds is hampered by more uncertainty. Nevertheless, for KN-62, docking scores clearly favor binding poses involving the intersubunit allosteric pocket in agreement with the effects of D92A, F103A, T90V, and V312A mutations on KN-62 inhibition (Supplemental Table 3). The two “quinoline arms” of KN-62 are almost identical, hence the precise positioning of KN-62 in the allosteric site is ambiguous. A common feature of alternative poses is the phenyl arm placed deep in the intersubunit allosteric pocket between two P2X7R subunits, and one of the quinoline arms occupying the top of intersubunit allosteric binding site, whereas the second quinolone arm is pointing into a second of the intersubunit allosteric binding sites accessed via the central cavity (Fig. 5). In this pose, F103 and V312 are part of the pocket accommodating the phenyl arm of KN-62, while one quinolone arm is in proximity to F88.

For calmidazolium, both stereoisomers were used in docking. The best docking scores were for poses involving the intersubunit allosteric binding site, but with virtually no difference found for the *R* and *S* stereoisomers (Supplemental Table 3). Interestingly, the representative poses for the main clusters of both stereoisomers are remarkably similar and occupy the same space, with the dichlorophenyl groups occupying the intersubunit allosteric binding pocket while the imidazole and its chlorophenyl substituents sit in the central cavity. As found for KN-62 and calmidazolium, brilliant blue G binding poses occupy the intersubunit allosteric binding pocket and the central cavity. The representative docking pose of the main cluster shows no direct contact with F88, but F103 is in close proximity to the ethoxy-phenyl group which might explain why the F103A, but not F88A, mutation affects brilliant blue G antagonism.

Docking poses for KN-62, calmidazolium, and brilliant blue G all make use of the intersubunit allosteric binding pocket, in agreement with mutagenesis data, but also occupy the central cavity. A consequence of such arrangements is that binding of three antagonist molecules to the trimeric hP2X7R receptor in structurally equivalent binding modes would not be possible. This implies a different binding stoichiometry compared with AZ11645373, and/or involvement of additional, but different binding modes.

## Discussion

In the present study we have used chimeras, point mutations, and ligand docking to provide validated molecular models of the intersubunit allosteric binding modes for the P2X7R antagonists AZ11645373, KN-62, calmidazolium, ZINC58368839, and BBG. The work also identifies two “signature” mutations (D92A and F103A) that can be used to identify binding at the intersubunit allosteric pocket.

Our work provides an empirically tested model of AZ11645373 binding at the intersubunit allosteric antagonist pocket that has been identified by crystallization and mutagenesis studies (Karasawa and Kawate, 2016; Allsopp et al., 2017, 2018). This contrasts with previous in silico docking around F95 (and for KN-62) that proposed an alternative allosteric site centered at the inner vestibule at the “top” of the extracellular portion of the receptor proximal to the orthosteric site (Caseley et al., 2015). Phenylalanine 95 lines both the inner vestibule and the allosteric pocket. We now show that species variation at residue 312 contributes to AZ11645373 and KN-62 sensitivity. This residue lines the allosteric pocket, but not the inner vestibule, and hence points to the intersubunit allosteric pocket as the site of action. Additional mutants in the intersubunit allosteric pocket modify AZ11645373 sensitivity, supporting the docked allosteric pose. Species differences in P2X7R properties provide an additional test for models of AZ11645373 antagonist binding. hP2X7R-like AZ11645373 sensitivity could be introduced to the insensitive rat receptor by two “humanizing” residue substitutions at positions 95 (L95F) and 312 (A312V), strongly supporting the intersubunit allosteric binding site model. Although the reciprocal point mutations in hP2X7R did not remove antagonist action, they decreased sensitivity at the hP2X7R 30- to 100-fold. This suggests that both F95 and V312 exert dominant effects on AZ11645373 binding; if either of them is present the receptor shows inhibition. The effect of these two residues is also captured in ligand docking in which the rP2X7R poses show higher scores (weaker binding) compared with hP2X7R, providing further evidence for our proposed AZ11645373 binding pose.

ZINC58368839 had been originally tested as a result of screening a compound library against the P2X7R orthosteric site. The current study provides evidence that mutation in the intersubunit allosteric pocket can abolish ZINC58368839 action. The notion of binding to the intersubunit allosteric pocket is supported by ligand docking, as scores are more favorable and the suggested binding mode resembles binding poses characterized by X-ray crystallography (Karasawa and Kawate, 2016). In the proposed binding pose the hydrophobic cycloheptane group of ZINC58368839 sits deeply in the intersubunit allosteric pocket. This is in agreement with the finding that substituting cycloheptane with a phenyl group diminishes activity (Caseley et al., 2016), as the level of hydrophobic interactions would be reduced.

Brilliant blue G, KN-62, and calmidazolium are bigger in size than AZ11645373, ZINC58368839, and antagonists binding in the intersubunit allosteric pocket studied previously (Karasawa and Kawate, 2016; Allsopp et al., 2017, 2018), raising the question whether such binding poses might be possible for these compounds. The non-specific P2X receptor antagonist PPADS has two sulfate groups and a phosphate group, and in case of P2X1R binding in the proximity of the ATP binding site driven by the positively charged “cloud” around the orthosteric site has been established (Huo et al., 2018). This is in agreement with the finding that in hP2X7R the signature intersubunit allosteric mutants D92A, F103A, and V312A show no effect on PPADS potency. Brilliant blue G has two features in common with PPADS: 1) It has two negatively charged sulfate groups (though one of them is compensated by a positive iminium charge), and 2) they both have a noncompetitive mode of action resulting in a collapse in the concentration response curve to agonist with no change in EC_50_. However, an orthosteric mode seems improbable for brilliant blue G, as D92A, F103A, and V312A mutations decreased BBG action. For both KN-62 and calmidazolium, the characterization of “signature” mutants results in a similar pattern of effects, in which four or five the mutations abolish or significantly reduce antagonist activity (T90V for calmidazolium being the exception). These results provide strong evidence for an involvement of the intersubunit allosteric site in KN-62 and calmidazolium binding. Ligand docking proposes binding poses that involve the intersubunit allosteric site but also the central cavity. Such a binding mode is different from the three equivalent binding sites proposed for AZ11645373 and ZINC58368839, but this might also provide a structural narrative for the finding that brilliant blue G, KN-62 (consistent with previous characterization Michel et al., 2000), and calmidazolium had Hill slopes of <1, indicating either more than one mode of binding or negative cooperative binding. Mutagenesis data and ligand docking for brilliant blue G, KN-62, and calmidazolium predict binding poses for these three antagonists that use the intersubunit allosteric pocket but also expand into the central cavity. A consequence of this model is nonstoichiometric binding and negative cooperativity for any additional binding event.

Analysis of mutants of the intersubunit allosteric pocket in P2X7R now can be made for a range of antagonists (Karasawa and Kawate, 2016; Allsopp et al., 2017, 2018). Among the mutations that consistently show the largest effects are D92A and F103A. D92 is fully conserved among human P2XR paralogs. Although the D92 backbone atoms are lining the intersubunit allosteric pocket, its side chain is not directly part of it, suggesting an indirect effect. Molecular dynamics simulations indicated that the D92A mutation destabilizes the interaction between D92 and Y298, and probably changes the shape of the pocket (Allsopp et al., 2018). F103 is an aromatic residue in the middle of pocket with an alanine mutation having an effect on all P2X7R allosteric antagonists tested so far, suggesting that interaction with this residue is a key feature of binding to the allosteric site. These results highlight that a decrease in antagonist sensitivity at the D92A and F103A mutants is diagnostic of binding at the P2X7R intersubunit allosteric site, and extends the number of antagonists to eleven for which evidence is strong of binding to this site (Supplemental Table 8). Beyond the F103A and D92A mutations, there is an additional “fine structure” of mutations, in which mutations such as T90V have effects on some but not all antagonists. Elucidating the full structural basis for this “fine structure” is a challenge for future work.

## Abbreviations

AZ11645373: 3-[1-[[(3′-Nitro[1,1′-biphenyl]-4-yl)oxy]methyl]-3-(4-pyridinyl)propyl]-2,4-thiazolidinedione
BBG: brilliant blue G
Calmidazolium: 1-[*bis*(4-chlorophenyl)methyl]-3-[2-(2,4-dichlorophenyl)-2-(2,4-dichlorobenzyloxy)ethyl]-1H-imidazolium
KN-62: 4-[(2S)-2-[(5-isoquinolinylsulfonyl)methylamino]-3-oxo-3-(4-phenyl-1-piperazinyl)propyl] phenyl isoquinolinesulfonic acid ester
PPADS: Pyridoxalphosphate-6-azophenyl-2′,4′-disulfonate
P2XR: P2X receptor
ZINC5836883: *N*-cycloheptyl-*N*-methyl-2-(5-nitro-1H-indol-1-yl) acetamide
